# Resveratrol, a SIRT1 Activator, Ameliorates MK-801-Induced Cognitive and Motor Impairments in a Neonatal Rat Model of Schizophrenia

**DOI:** 10.3389/fpsyt.2020.00716

**Published:** 2020-07-24

**Authors:** Juan Niu, Yuquan Cao, Yongjuan Ji

**Affiliations:** ^1^ Psychological Clinic, The Affiliated Hospital of Qingdao University, Qingdao, China; ^2^ Rizhao Mental Health Center, Rizhao, China; ^3^ Department of Mental Health, Qingdao Women and Children’s Hospital, Qingdao, China

**Keywords:** resveratrol, MK-801, silent information regulator 1, brain derived neurotrophic factor, schizophrenia

## Abstract

**Background:**

In neonatal rats, MK-801 treatment generates schizophrenia-like symptoms. Resveratrol (RSV) is a phenolic compound and a potent neuroprotective agent. This research aimed to illustrate the effect of RSV on the amelioration of MK-801-induced cognitive and motor impairments in a neonatal rat schizophrenia model and the related potential molecular changes.

**Methods:**

Rats were administrated with MK-801, MK-801 + RSV (40 mg/kg), or MK-801 + RSV (80 mg/kg). Motor learning, coordination, locomotor and exploratory activity, and spatial memory were measured by rotarod test, pen field test, and Morris water maze test. Relative protein levels were analyzed by Western blot and ELISA. mRNA levels were shown by qRT-PCR.

**Results:**

In the hippocampus of MK-801-induced schizophrenia rat model, RSV enhanced silent information regulator 1 (SIRT1) and brain derived neurotrophic factor (BDNF) expression and alleviated oxidative stress. Motor perturbations and learning impairments by MK-801 treatment were ameliorated by the administration of RSV.

**Conclusion:**

In conclusion, RSV showed neuroprotective effect on MK-801-induced schizophrenia rat model through regulating SIRT1 and downstream BDNF expression in the hippocampus.

## Introduction

As a mental disorder, schizophrenia has a lifetime prevalence of 0.3–0.66% throughout the world (1). The pathogenesis of schizophrenia involves several different neurotransmitter systems and enzymes (2). Based on previous research, patients with schizophrenia manifest several cognitive symptoms, including decreased memory, decreased attention and reasoning skills (3). The incidence of schizophrenia increases during adolescence and the peak age of schizophrenia onset is from 15 to 30 years (4). In schizophrenia patients, approximately 20% of individuals suffer the disorder before 18 years old (5). It is reported that the early pathogenesis of schizophrenia is correlated with long-term poor prognosis, high suicidality, and social defect (6, 7).

MK-801 is an effective and non-competitive N-methyl-[D]-aspartate (NMDA) receptor antagonist that influences the nervous system (8). MK-801 administration in rats can induce several behavioral changes, including ataxia, cognitive deficit, hyperlocomotion, and stereotyped movement, which are also symptoms caused by schizophrenia (9). Schizophrenia-caused alternation of neurotrophic factors and neurotransmitter systems can also be mimicked by MK-801 administration (10). Therefore, MK-801 treatment is employed for the induction of some schizophrenia-like symptoms in rats.

Resveratrol (RSV) is a phenolic compound which is reported to be a potent neuroprotective agent (11). Recent research has shown that RSV is an effective silent information regulator 1 (SIRT1) activator (12). SIRT1 participates in various biological procedures, including neuroprotection, mitochondrial function, oxidative stress, and autophagy (13). Based on these functions, SIRT1 is involved in the pathogenesis of neurodegenerative disorders (14). Through enhancing the expression of cAMP response element-binding protein (CREB) and brain derived neurotrophic factor (BDNF), SIRT1 promotes hippocampal-dependent memory and learning abilities in a mouse model (15). BDNF is extensively studied to be an important factor of schizophrenia pathology or pathogenesis (16). Significantly downregulated BDNF expression is associated with symptoms in schizophrenia, including cognitive disorders (17). Previous study has already demonstrated that the administration of RSV in mice can generate anxiolysis and anti-psychotic effects (18).

In this research, we aimed to illustrate the function of RSV in the amelioration of MK-801-induced cognitive and motor impairments in a neonatal rat schizophrenia model and the related potential molecular changes.

## Methods

### Animals

Male Sprague–Dawley rats were kept in standard conditions. All animal procedures were approved by Qingdao Women and Children’s Hospital. Animals were randomly divided into four groups (n = 10 each): (1) control group; (2) MK-801 (1 mg/kg), (3) MK-801 (1 mg/kg) and RSV (40 mg/kg); and (4) MK-801 (1 mg/kg) and RSV (80 mg/kg). MK-801 (1 mg/kg) and RSV treatments were given intraperitoneally on postnatal days (P) 7–14 once a day. Biochemical analysis was carried out at P14 with 10 rats in each group. Behavior experiments were performed at P55 with 10 rats in each group. RSV was purchased from Solarbio (Beijing, China) and MK-801 from Sigma-Aldrich (St. Louis, MO, USA). Drug were dissolved in saline solutions.

### QRT-PCR

The total RNA of hippocampal tissue was extracted by Trizol (Invitrogen, Carlsbad, CA, USA). cDNA was reverse-transcribed from total RNA through First Strand cDNA Synthesis Kit (Sigma, St. Louis, MO, USA). SYBR Green Real-Time PCR Master Mixes (ThermoFisher, Waltham, MA, USA) was employed to perform real-time PCR. Glyceraldehyde-3-phosphate dehydrogenase (GAPDH) was used as the internal control. Primers purchased from Ruibio BiotechCo., Ltd (Beijing, China) were:

SIRT1 F: TGACCTCCTCATTGTTATTGGG
SIRT1 R: GGCATACTCGCCACCTAACC
BDNF F: AGCTGAGCGTGTGTGACAGTAT
BDNF R: CCGAACATACGATTGGGTAGTT
GAPDH F: CAAGGTCATCCATGACAACTTTG
GAPDH R: GTCCACCACCCTGTTGCTGTAG

### ELISA

The serum levels of BDNF and SIRT1 were quantified by relative ELISA kit. BDNF: Boster EK0308 size 96T (Boster-Bio, CA, USA). SIRT1: USCN E94912Ra (Uscn Life Science Inc., GA, USA). The concertation of relative protein was calculated from OD values through standard curves.

### Oxidative Stress Measurement

Malondialdehyde (MDA) level in hippocampal tissue was measured by MDA assay kit (TBA method) (Nanjing Jiancheng, Nanjing, China). Glutathione (GSH) level was measured by reduced GSH assay kit (Nanjing Jiancheng). The activity of total superoxide dismutase (T-SOD) was evaluated by T-SOD assay kit (Hydroxylamine method) (Nanjing Jiancheng). The activity of catalase (CAT) was evaluated by CAT assay kit (Nanjing Jiancheng). All the kits were used based on the manufacturer’s instructions.

### Western Blot

Hippocampal tissues were lysed by RIPA buffer (Beyotime, Shanghai, China). Lysate was centrifuged at 1,200*g* for 10 min at 4°C and the supernatant was collected as the protein sample. Protein samples were separated by 10% SDS/PAGE and transferred to nitrocellulose membrane. After blocking, membranes were incubated with relative primary antibodies, including SIRT1 (Abcam, Cambridge, UK), CREB (Cell Signaling Technology, Danvers, MA, USA), Phospho-CREB (Cell Signaling Technology), BDNF (Abcam), and GAPDH (Abcam) at 4°C overnight. The membranes were washed by TBST and then incubated with relative secondary antibodies for 1 h at room temperature. Protein blots were visualized by ECL Substrate Kit (Abcam, Cambridge, UK). The gray value of the band was calculated by ImageJ.

### Rotarod Test

The rotarod test equipment was an accelerating rotating rod and used for evaluating the motor coordination and balance of rats (19). In this experiment, the minimum speed was 10 revolutions per minute (rpm) and increased linearly to the maximum speed of 60 rpm during each trial which lasted 5 min. Each rat had three trials and the interval was 30 min. The rats’ motor coordination and balance were shown by latency to fall.

### Open Field Test

This equipment was a 90 × 90 × 30 cm square built with plexiglass. The square was separated into 16 equal square regions. The experiment was executed in a room with diminished light and sound, provided with homogenous and indirect illumination. Rats were allowed for free exploration in equipment for 5 min. Ethovision software (Noldus Information Technology) was employed for recording and analyzing the behaviors of rats. Behavioral indices measured in this experiment were shown as follows: moved distance, velocity, and rearing number.

### Morris Water Maze Test

The Morris water maze test is a reliable paradigm of cognitive function, which can test learning and memory deficits (20). This equipment was a circular tank with 45 cm depth and 140 cm diameter with 22–24°C water. A 15 cm wide and 35 cm high hidden platform was placed 1.5 cm beneath the water. In the testing room, different visual cues on the wall surrounded the equipment and the relative location was unchanged. The equipment was separated into four equal quadrants. Rats were placed randomly in one quadrant. A camera was located above the center of apparatus and recorded the behavior of rats during the experiment. Video-tracking system software (Ethovision) was employed for evaluating and analyzing relative parameters including escape latency and traveled distance.

Training session was composed of three blocks with 30 min interval between each block. Each session had four consecutive trials. In each trial, rats were placed randomly in a quadrant and allowed to swim to find the hidden platform for 1 min. Rats were allowed to stay on the platform for 20–30 s and then were caged for 20–30 s before the next trial. Two hours after training, a 60 s probe trial was performed in this equipment without platform to evaluate the spatial memory retention.

### Statistical Analysis

SPSS version 22.0 was employed for data analyses. Data from rotarod test and Morris water maze test were analyzed by repeated-measures ANOVA and Bonferroni post-hoc test. Other data were analyzed by one-way ANOVA. Data were presented as mean ± SD. P <0.05 was considered to be significant difference.

## Results

### RSV Enhances the Expressions of SIRT1 and BDNF in the Serum and Hippocampus of MK-801-Induced Schizophrenia Rat Model

**Figure 1:**
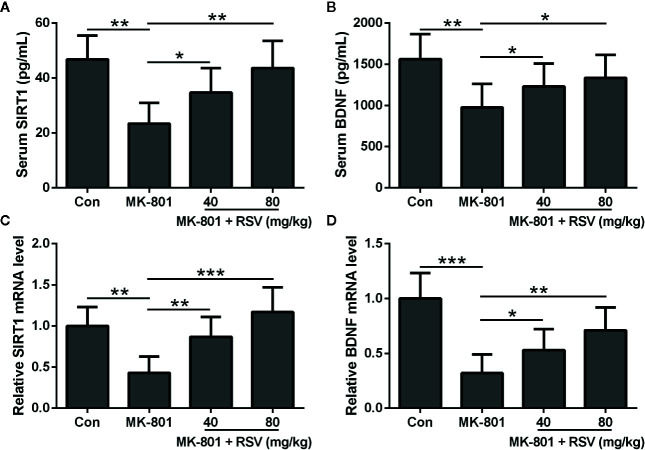
RSV ameliorates the expressions of SIRT1 and BDNF in rat serum and hippocampus. ELISA was used to measure the concentration of SIRT1 **(A)** and BDNF **(B)** in serum. QRT-PCR was used to analyzed the mRNA levels of SIRT1 **(C)** and BDNF **(D)** in hippocampus. GAPDH was set as a loading control and the relative expressions were normalized to control group. n = 6 in each group. Data were presented as mean ± SD. F ratios: **(A)** 11.39 (3.00, 17.43), **(B)** 5.783 (3.00, 21.06), **(C)** 13.33 (3.00, 18.60), **(D)** 16.40 (3.00, 12.70). *p < 0.05, **p < 0.01, and ***p < 0.001 between the indicated groups.

In this research, animals were divided into four groups: (1) control group; (2) MK-801 (1 mg/kg) group; (3) MK-801 (1 mg/kg) and RSV (40 mg/kg) group; and (4) MK-801 (1 mg/kg) and RSV (80 mg/kg) group. In our preliminary experiment, we observed that the treatment of 80 mg/kg resveratrol had no effects on the cognitive and motor functions of rats. We first examined the protein levels of SIRT1 and BDNF in the serum after respective drug treatment. As shown in **Figures 1A, B**, the administration of MK-801 significantly declined the protein levels of SIRT1 and BDNF in the serum. The simultaneous administration of RSV with MK-801 significantly alleviated the MK-801-reduced SIRT1 and BDNF protein levels in the serum in a dose-dependent manner (**Figures 1A, B**). We also evaluated SIRT1 and BDNF mRNA levels in the rat hippocampus tissues. Based on the result of qRT-PCR, SIRT1 and BDNF mRNA levels in the hippocampus showed the same trends as their serum levels after treatment with respective drugs (**Figures 1C, D**). Therefore, the administration of RSV ameliorated the MK-801-inhibited expression of SIRT1 and BDNF in the serum and hippocampus of model rats.

### RSV Enhances CREB Phosphorylation and Elevates SIRT1 and BDNF Protein Levels in the Hippocampus of MK-801-Induced Schizophrenia Rats

**Figure 2:**
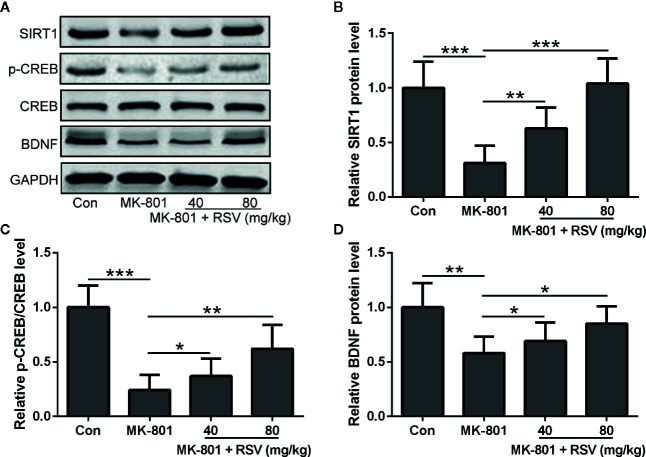
Effect of RSV on the protein expression of SIRT1 and BDNF and CREB phosphorylation in the hippocampus of MK-801 treated rats. **(A)** Western blot was used to measure the protein expression of SIRT1, p-CREB, CREB, and BDNF in hippocampus. GAPDH was used as loading control. Relative expression was normalized to control group. n = 6 in each group. Data were presented as mean ± SD. F ratios: **(B)** 21.96 (3.00, 19.71), **(C)** 26.80 (3.00, 15.08), **(D)** 8.625 (3.00, 11.37). *p < 0.05, **p < 0.01, and ***p < 0.001 between the indicated groups.

Since SIRT1, BDNF, and CREB were shown to be critical for neural survival, these proteins in the rat hippocampus were detected by Western blot (**Figure 2A**). In the rat hippocampus, the protein level of SIRT1 was reduced by MK-801 treatment and significantly alleviated by the administration of RSV (**Figure 2B**). Depressed CREB phosphorylation in MK-801-induced schizophrenia rats was also ameliorated by the treatment of RSV (**Figure 2C**). As shown in **Figure 2D**, the administration of RSV also alleviated the MK-801-reduced protein level of BDNF in the rat hippocampus.

### Oxidative Stress in the Hippocampus of MK-801-Treated Rats Was Inhibited by the Administration of RSV

**Figure 3:**
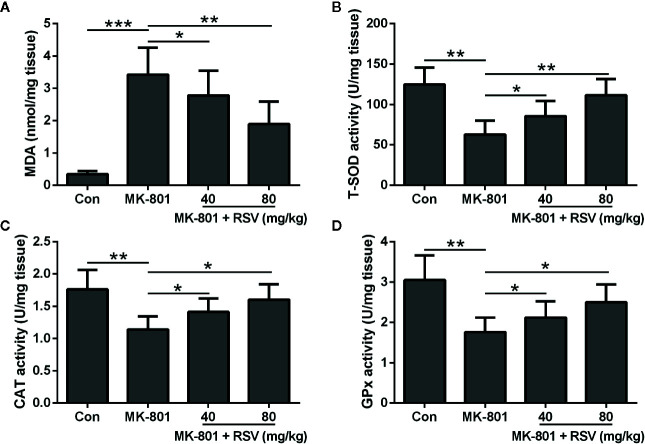
Effect of RSV on the levels of oxidative stress in the hippocampus of MK-801 treated rats. MDA levels **(A)**, T-SOD activity **(B)**, CAT activity **(C)**, and GPx activity **(D)** were detected in the hippocampus. n = 6 in each group. Data were presented as mean ± SD. F ratios: **(A)** 31.98 (3.00, 20.77), **(B)** 16.36 (3.00, 17.38), **(C)** 9.818 (3.00, 21.12), **(D)** 11.39 (3.00, 13.45). *p < 0.05, **p < 0.01, and ***p < 0.001 between the indicated groups.

To demonstrate the function of RSV in ameliorating MK-801-induced schizophrenia in rats, oxidative stress in the hippocampus was evaluated. **Figure 3A** showed that the MK-801-elevated MDA levels in the rat hippocampus were significantly decreased by RSV treatment. Comparisons between different groups indicated that CAT, T-SOD, and GPx activities were dramatically enhanced after RSV treatment in MK-801-treated rat hippocampus (**Figures 3B–D**). In summary, RSV treatment abolished the MK-801-induced oxidative stress.

### RSV Treatment Enhances the Motor Learning and Coordination of MK-801-Treated Rats

**Figure 4:**
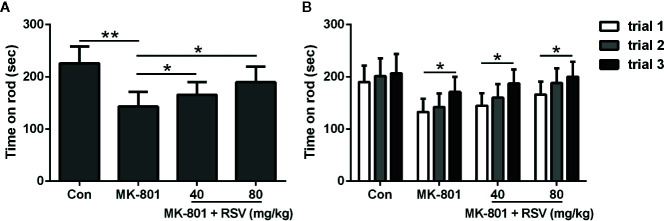
Effect of RSV on MK-801-induced deficits in the rotarod test. Mean latency of three trials of staying on the rod **(A)** and falling time in three successive trials **(B)** were compared. n = 10 in each group. Data were presented as mean ± SD. F ratios: **(A)** 12.28 (3.00, 17.91). *p < 0.05, **p < 0.01 between the indicated groups.

In **Figure 4A**, the mean latency of three trials of staying on the rod was shown. When compared with rats in the control group, rats treated by MK-801 spent a significantly shorter time on the accelerating rotarod. Meanwhile, simultaneous administration of MK-801 and RSV dramatically extended the time on rod. Task acquisition in different groups were shown in **Figure 4B**, which showed significant improvement between trial 3 and trial 1 in MK-801 group, MK-801 + RSV (40 mg/kg) group, and MK-801 + RSV (80 mg/kg) group, except the control group. These data indicated that RSV had effect in the improvement of motor performance in rotarod task in MK-801-treated rats.

### RSV Treatment Regulates the Locomotor and Exploratory Activity of MK-801-Treated Rats

**Figure 5:**
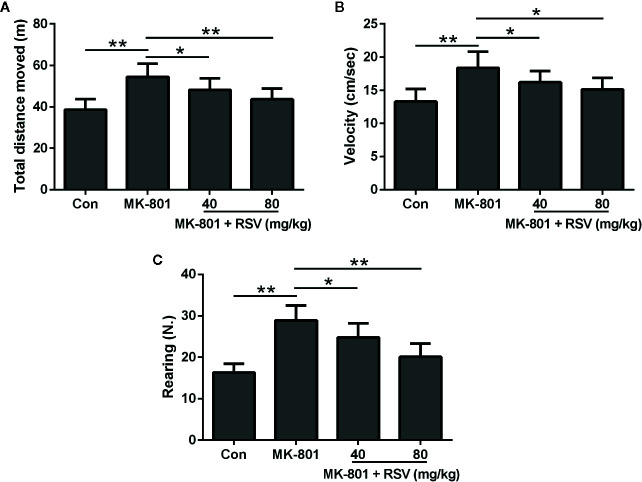
Effect of RSV on the locomotor and exploratory activity of rats exposed to MK-801 in the open field test. Total distance moved **(A)**, velocity **(B)**, and rearing number **(C)** were evaluated. n = 10 in each group. Data were presented as mean ± SD. F ratios: **(A)** 11.86 (3.00, 20.10), **(B)** 9.394 (3.00, 18.85), **(C)** 24.63 (3.00, 21.22). *p < 0.05, **p < 0.01 between the indicated groups.

**Figure 5A** showed the total distance moved during the open field test. Rats treated by MK-801 had a significantly longer distance than those in the control group. However, the distance was shortened by RSV treatment in MK-801-treated rats. We also evaluated the velocity of rats in the open field test (**Figure 5B**). Based on statistical analysis, the velocity increased by MK-801 administration was significantly decreased by RSV treatment in rats. The number of vertical locomotor activity (rearing) was also measured to indicate the exploratory behavior of rats (**Figure 5C**). Based on statistical analysis, the rearing number increased by MK-801 administration was significantly decreased by RSV treatment in rats. Therefore, RSV inhibited the hyperactive effect of MK-801 in rats.

### RSV Treatment Enhances the Learning and Memory Abilities of MK-801-Treated Rats

**Figure 6:**
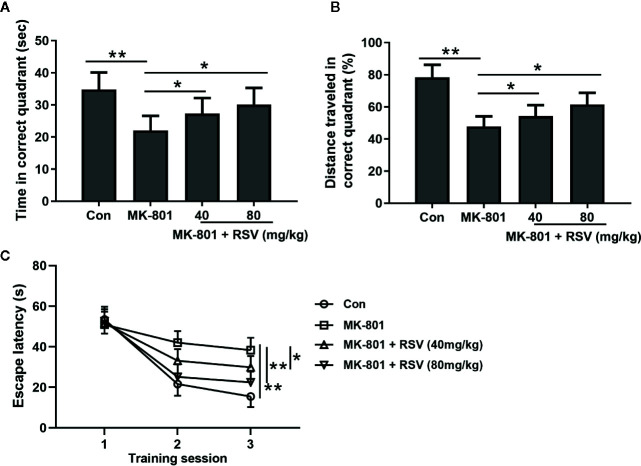
Effect of RSV on spatial learning of rats exposed to MK-801 in the Morris water maze task. Time spent in the target quadrant **(A)**, distance traveled in the target quadrant **(B)** and escape latencies during the three training sessions **(C)** were compared. n = 10 in each group. Data were presented as mean ± SD. F ratios: **(A)** 9.172 (3.00, 17.55), **(B)** 28.06 (3.00, 20.34). *p < 0.05, **p < 0.01 between the indicated groups.

The effects of RSV on the spatial learning of MK-801-treated rats were measured in the Morris water maze. In this experiment, the memory performance of rat was shown by the time spent (**Figure 6A**) and the percentage of traveled distance in the correct quadrant (**Figure 6B**). When compared with rats in the control group, the MK-801-treated rats had significantly shorter time and traveled distance in the correct quadrant. However, the administration of RSV significantly elevated the time and traveled distance. Escape latency was also measured in this experiment. As shown in **Figure 6C**, in later training stages, mean escape latency was significantly shortened by the administration of RSV. These results illustrated that the inhibited memory performance in MK-801-treated rats was reversed by RSV treatment.

## Discussion

In this research, we illustrated the symptoms of cognitive and motor impairments generated by MK-801 administration in a neonatal rat model and demonstrated the preventive function of RSV treatment in these symptoms. MK-801-treated rats showed locomotor agitation, balance perturbation, and spatial learning and memory disturbances. RSV treatment in the schizophrenia neonatal rat model efficiently prevented cognitive and motor impairments. Besides the generation of cognitive and motor deficits, this research also showed that the administration of MK-801 inhibited the expression levels of SIRT1 and BDNF and decreased the CREB phosphorylation level in the hippocampus. These protein alterations triggered by MK-801 administration in the neonatal rat model were all normalized by RSV treatment.

It has been reported that, postnatal day 7 to day 14 is a crucial period for the development of nervous system and the expression of NMDA receptor in rodents (21). Signaling pathways which contain NMDA receptor have critical functions in learning, memory, cognition, locomotion, and synaptic plasticity (22–25). Since the administration of NMDA antagonists, including MK-801, during the first three weeks after birth can induce several core symptoms of schizophrenia, these antagonists are widely used in the construction of rodent schizophrenia models (26). Systemic NMDA antagonist administration during several weeks after birth induces defects in locomotor activity and cognitive function at adulthood (27). In this research, the neonatal rat schizophrenia model was induced by MK-801 administration from P7 to P14.

In the MK-801-induced rat schizophrenia model, the hippocampus is shown to be the most sensitive part in the brain and its structure plasticity is impaired (28). In the hippocampus, the expression of BDNF has an essential function in regulating the length and branching of dendritic cells through the activation of its downstream proteins (29). The decreased peripheral concentration of BDNF is widely observed in patients with schizophrenia (12). Several different studies have reported the correlation between the pathogenesis of schizophrenia and the abnormal expression of BDNF (30, 31). In rats, the cognitive deficits generated during NMDA antagonist administration was also triggered by depressed BDNF expression in the hippocampus (32). In this research, we confirmed that the administration of MK-801 inhibited the expression of BDNF in the rat hippocampus. However, the treatment of RSV rescued the decreased BDNF expression level in a dose-dependent manner. Another research showed that MK-801 administration also inhibited the phosphorylation of CREB in the rat hippocampus (33). CREB is the upstream transcription factor of BDNF and inhibited CREB phosphorylation downregulates BDNF expression (34). In the neonatal rat schizophrenia model induced by MK-801 administration, we observed the inhibited phosphorylation of CREB in the hippocampus. The depressed CREB phosphorylation was also restored by the administration of RSV.

SIRT1 is a crucial epigenetic modulator in the brain, especially in the hippocampus (35). SIRT1 participates in the differentiation and development of cognitive function-related neurons and its abnormal expression is connected with cognitive domain damage (36). The inhibited expression of SIRT1 impairs cognitive function and depresses the expression of BDNF (37). Recent research has shown that RSV is an effective SIRT1 activator (12). In this research, the depressed expression of SIRT1 in the neonatal rat schizophrenia model was elevated by the administration of RSV. Therefore, RSV treatment ameliorates the depressed CREB/BDNF pathway in the neonatal rat schizophrenia model through enhancing SIRT1 expression.

In recent years, the accumulation of reactive oxygen species (ROS) is found to contribute to the pathogenesis of schizophrenia (38). The accumulation of ROS and the abnormal function of anti-oxidant enzymes generate enhanced oxidative stress in schizophrenia (39). MDA levels in schizophrenia patients are increased during the pathogenesis (40). In the hippocampus of MK-801 treated rats, MDA level was also elevated. Another study has demonstrated that the altered activities of SOD, CAT, and GPx damage lipids and proteins in a schizophrenia rat model (41). In this research, the activities of SOD, CAT, and GPx were also inhibited by the administration of MK-801. However, the treatment of RSV significantly reduced MDA level and enhanced the activities of SOD, CAT, and GPx in the hippocampus of MK-801-treated rats. These results showed that the enhanced oxidative stress in the schizophrenia rat model was alleviated by the treatment of RSV.

Rotarod test is reported to be a reliable tool for evaluating motor skill learning of the rodents (19). The imbalance in the rotarod test is considered to be a “non-dopamine dependent” motor function which can be triggered by the administration of NMDAR antagonists such as MK-801 (42). The results from rotarod test showed that RSV administration rescued the MK-801-induced balance disturbance in rats. The open field test is a widely used ethological test for assessment of anxiety-like behaviors in rodents (43). Previous research has demonstrated that moved distance, velocity, and rearing number are increased by MK-801 treatment in rats (44). In this research, we observed the same phenomenon in MK-801-treated rats during the open field test. The combination of RSV with MK-801 dramatically decreased the total distance, velocity, and the number of rearing. Our data illustrated the effect of RSV on the locomotor and exploratory activity in the MK-801-induced schizophrenia rat model. The Morris water maze test is a reliable method for the measurement of cognitive functions of the rodents (45). In this study, this experiment was explored for evaluating the potential function of RSV on spatial learning and memory ability in the MK-801-induced schizophrenia rat model. According to our results, the administration of RSV ameliorated the spatial memory deficit generated by MK-801 treatment in rats. Therefore, in the neonatal rat schizophrenia model induced by MK-801 administration, RSV treatment was beneficial for the amelioration of cognitive and motor impairments. However, the neuroprotective effect of RSV in MK-801-induced schizophrenia rat model was limited. RSV could only marginally reverse these cognitive and motor impairments but could not completely rescue them.

This research still had some limitations that should be mentioned. The function of RSV in alleviating oxidative stress in the schizophrenia rat model was demonstrated, but the underlying molecular mechanism was not clear. Since various neurotransmitters and receptors participate in the regulation of motor coordination and learning ability, further studies are required for elucidating other potential molecular mechanisms of RSV in schizophrenia. In this study, there was no RSV alone control group, which was necessary in order to conclude that the changes observed could be caused by the action of RSV on these parameters rather than induced by MK-801. In this research, biochemical analyses were carried out at P14 and behavior experiments were performed at P55, based on the assumption that SIRT1/CREB/BDNF signaling pathway in hippocampus activated by RSV at P15 would be maintained through to P55. This long-term effect of RSV contributed to its neuroprotective effect in MK-801-induced schizophrenia rat model. However, another possibility also should be considered: the long-term RSV neuroprotective effect might have no association with SIRT1/CREB/BDNF signaling pathway but influence other schizophrenia-related signaling pathways.

## Conclusion

In summary, RSV showed a neuroprotective effect in the MK-801-induced schizophrenia rat model through activating the SIRT1/CREB/BDNF signaling pathway in the hippocampus.

## Data Availability Statement

The raw data supporting the conclusions of this article will be made available by the authors, without undue reservation.

## Ethics Statement

The animal study was reviewed and approved by Qingdao Women and Children’s Hospital. Written informed consent was obtained from the owners for the participation of their animals in this study.

## Author Contributions

YJ wrote the first draft of the study protocol and survey. JN and YC contributed to revisions of the protocol, including survey development. JN contributed to recruitment and data collection and wrote the first draft of the manuscript. YJ contributed to revising the second draft. All authors contributed to the article and approved the submitted version.

## Conflict of Interest

The authors declare that the research was conducted in the absence of any commercial or financial relationships that could be construed as a potential conflict of interest.
